# A Randomised, Double-Blind Pilot Study of Enzyme-Potentiated Desensitisation for Prophylaxis of Large Local Reactions to Mosquito Bites 

**DOI:** 10.1155/2012/106069

**Published:** 2012-03-22

**Authors:** S. Berkovitz, N. Hill, M. Radcliffe, G. Ambler

**Affiliations:** ^1^Royal London Hospital for Integrated Medicine, University College London Hospital NHS Foundation Trust, 60 Great Ormond Street, London WC1N 3HR, UK; ^2^Disease Control & Vector Biology Unit, Faculty of Infectious and Tropical Diseases, London School of Hygiene & Tropical Medicine, Keppel Street, London WC1E 7HT, UK; ^3^Sarum Road Hospital, Allergy Clinic, Sarum Road, Winchester SO22 5HA, UK; ^4^Joint UCLH/UCL Biomedical Research Unit, Ground Floor, Rosenheim Wing, 25 Grafton Way, London WC1E 6DB, UK

## Abstract

*Primary Objective*. To test the hypothesis that two injections of enzyme-potentiated mosquito antigen significantly reduce the size of experimental mosquito bites in participants with LLR-MB. *Design*. Randomised, double-blind, placebo-controlled, parallel group comparison over 3 months. *Setting*. Hospital outpatient clinic. *Participants*. Fifty adult participants of both sexes. *Interventions*. Two injections of mosquito antigen or matching placebo, 6 weeks apart. *Main Outcome Measures*. Early (1 hour) and late (24 hours) mean square root of erythema area (SREA) following controlled mosquito bite with the second bite given at least 6 weeks following the final injection. *Results*. At 1 hour, mean SREA was slightly higher in the EPD group compared to placebo after adjusting for baseline values (0.46, 95% CI −6.11 to 7.03), but this was not statistically significant (*P* = 0.89, ANCOVA analysis); neither were the results at 24 hours (−2.58, 95% CI −11.73 to 6.57) (*P* = 0.57). The proportion of participants experiencing a decrease in wheal size at 1 or 24 hours was similar between groups. *Conclusions*. EPD was not demonstrated to be effective for immediate or delayed LLR-MB. Methodological problems included a high variability in LLR-MB between subjects, suggesting that a crossover design should be used in future.

## 1. Introduction

Large local reactions to mosquito bites (LLR-MB) are common and sometimes distressing and may be immediate, delayed, or both [1]. The mechanism is still controversial, with some authors favouring an IgE- or IgG_4_-mediated allergic process [2, 3] and others a lymphoproliferative process [4]. Although clinical studies have demonstrated the effectiveness of antihistamines as an acute treatment for LLR-MB [5], conventional specific immunotherapy has not been generally used. This is presumably because, in contrast to wasp or bee stings, mosquito bites almost never cause life-threatening reactions [6].

Enzyme-potentiated desensitisation (EPD) is a therapeutic technique in use for 30 years [7–10] in which low-dose allergens (10^−12^ mmol/L, comparable to skin prick test doses) are given intradermally in combination with the enzyme beta-glucuronidase, which is used to enhance the desensitising effect of the low-dose allergen. Prospective audit has demonstrated EPD to be safer than conventional immunotherapy [11] with no reports of systemic allergic reactions. Personal clinical experience and an unpublished survey of EPD users suggested beneficial results of an EPD mosquito antigen product in clinical use for several years for LLR-MB. The relative safety and apparent effectiveness of EPD as a preventative approach prompted the present study.

Several small but poorly reported RCTs using EPD based on mixed inhalant allergens suggested effectiveness in seasonal allergic rhinitis due to grass pollen [12–15] and childhood house dust mite allergy [16], although a larger and more fully reported clinical trial failed to confirm the results for seasonal allergic rhinitis [17]. Single studies have shown positive results using mixed food allergens for childhood attention deficit disorder [18] and childhood migraine [19]. The putative mechanism of action of EPD is unclear. It does not have an effect on B-cell immunity or generate “blocking” antibody nor does it alter skin prick test results or specific IgE levels [16]. However, it has been shown to alter cytokines involved with the immune response such as IL-6 and IL-10 [20].

The objective of the study was to test the hypothesis that two injections of EPD mosquito antigen would result in a reduced erythema area after controlled mosquito bite challenge in a group of adult sufferers of LLR-MB, when compared against a similar group given placebo injections. As a pilot study, we also aimed to test the methodology and provide estimates for a sample size calculation to power a definitive study if the results proved encouraging.

## 2. Materials and Methods

The study was conducted in accordance with the Declaration of Helsinki [21]. The study was conducted with the understanding and the written informed consent of the subjects. The study was approved by the Joint University College London Hospitals/University College London Ethics Committee.

50 participants were recruited in London from a database kept by one of the authors (N. Hill) of LLR-MB sufferers who had participated in previous clinical studies of topical antibite agents. Most were employees at the London School of Hygiene and Tropical Medicine. Mosquito challenge was performed there by NH and EPD injections were performed at the Royal London Homeopathic Hospital outpatient department by S. Berkovitz. Participants were recruited from March to July 2003 and were not followed up beyond their final mosquito exposure, the last of which was performed in January 2004.

In order to be candidates for inclusion, participants had to be aged between 18 and 65, of either sex, with a diagnosis of LLR-MB determined by (1) a history of large local reaction after insect bite identified as mosquito by participants, (2) mean erythema diameter (ED) of 20 mm or greater within 24 h of first experimental challenge to mosquito bite (for details see below). They were not admitted to the study if any of the following criteria were present: (1) previous treatment with EPD, (2) treatment with conventional immunotherapy in the last 5 years, (3) history of anaphylaxis or laryngeal oedema, (4) moderate-to-severe asthma (as judged by causing nocturnal waking, emergency hospital attendance in last twelve months, or need for systemic steroid treatment in last twelve months), (5) currently pregnant or breast feeding, (6) severe skin disease or pigmentation making the measurement of erythema difficult (this excluded participants of Negroid or other dark skin type), (7) chronic severe urticaria or dermatographism, (8) use of regular oral antihistamine, non-steroidal anti-inflammatory drugs, oral steroids, or immunosuppressants, (9) concomitant serious illness or cardiovascular or respiratory disease requiring specialist care or drug treatment (other than essential hypertension or mild asthma); or (10) any other condition that in the investigators' opinion excluded the participant on grounds of safety.

Participants were randomised to receive two injections of mosquito antigen or placebo at an interval of six-to-eight weeks, the first no less than a week after the first mosquito exposure. Blocked randomisation was performed with random block sizes with the investigators blinded to the allocation list. Individualised EPD vials were kept refrigerated or in a thermos flask filled with iced water. EPD was supplied as sterilised individual vials containing mosquito antigen (extract of crushed laboratory-bred female *Aedes Aegypti* mosquito heads in buffer solution, containing approximately 120 fg of salivary gland per 0.04 mL) or placebo (an identical buffer solution without mosquito antigen). 0.04 mL of mosquito antigen or placebo was drawn up by the Gilman pipette and mixed with 0.01 mL of beta-glucuronidase (Seravac, 1500–2000 Fishman units/mL). The resulting 0.05 mL was drawn up in an insulin syringe and administered by intradermal injection into the flexor aspect of the forearm. All EPD vials were prepared by McEwen Laboratories, Pangbourne (details of the method of preparation are available from S. Berkovitz) and administered by S. Berkovitz who had several years' experience in the technique. Participants waited one hour after the first injection and half an hour after the second injection to ensure the absence of immediate adverse effects. They were asked to not to observe the immediate reaction to maintain blinding. They were given a questionnaire to post back after 24 hours to ascertain any delayed adverse effects and to report the presence and size of any swelling at the injection site (a possible cause for unblinding) and any itching.

The primary outcome measure was the square root of erythema area measured at 1 hour (T1) and 24 hours (T24) after mosquito exposure. A secondary outcome measure was the degree of itching at T0 and T24. Erythema area has been used by N. Hill in previous studies of topical antipruritics for mosquito bites and has been found to be a reliable measure. The square root transformation was used to satisfy statistical assumptions (normality).

Participants were all subjected to an initial mosquito exposure to confirm their eligibility for the study and again six-to-eight weeks following the second EPD injection. The method was as follows. Female (3-4 day old) *Aedes aegypti *mosquitos were starved for 24 hours prior to use. Per daily period, the mosquitoes were isolated singly in small plastic tubes closed by netting to prevent escape whilst enabling them to feed through the netting.

The participants' forearms were cleaned with 70% ethanol and allowed to dry. Each subject was exposed to a single *Aedes aegypti* mosquito bite. A mosquito tube was secured on the midpoint of the nondominant forearm and allowed to feed for 10 minutes. At the end of this period, the feeding status of the mosquito (fed, partially fed, unfed) was noted. If a mosquito was not observed to be feeding after 5 minutes, it was replaced by another. In addition, the number of probes (the places where the mosquito penetrates the skin to find a good blood source) was recorded, since saliva could be injected even if no blood is extracted.

The diameter of erythema was measured with a digital calliper (Mitutoyo Digimatic Calliper, 500-311). Erythema length and width were defined as the maximum diameter and orthogonal diameter, respectively, (in millimetres). Erythema area (EA) was calculated as the area of an ellipse with these diameters (in millimetres squared). If there was more than one erythematous bite, the largest was used for the measurement. All calliper measurements were performed by the same person throughout the study. Diameter measurements were made at 15 minutes (T0), 1 hour (T1), 24 hours (T24).

Participants were not permitted any symptomatic medication following mosquito exposure until 24 hours after exposure (the time of the final measurement of erythema diameter). They were allowed to use topically applied ice packs for symptomatic relief. Regular medication was permitted as usual.

With no previous studies of EPD or similar treatments in this condition on which, to base a power calculation, an approximate calculation, using ANCOVA methodology, was performed using data from a similar group of participants in a previous study of a mosquito repellent. To have an 80% chance of detecting as significant using a two-sample *t*-test (at the 5% level), a 45% reduction in erythema area after verum treatment with no change in the placebo group, with an assumed baseline mean area of 1206 mm^2^ and a standard deviation of 772 mm^2^, 33 participants were required in each group. We decided to recruit 25 participants in each group since we were not performing a definitive study. However we note that the latter sample size actually has 94% power to detect such as difference using analysis of covariance (ANCOVA), assuming a correlation of 0.7 between the pretreatment and posttreatment responses and no loss to followup. ANCOVA was used to analyse the results on an intention-to-treat basis, comparing the posttreatment wheal size adjusted for the pretreatment wheal size between EPD and placebo.

Randomisation to verum or placebo groups was performed by the trial statistician using block randomisation with blocks of random size. The code was posted to McEwen Laboratories in Pangbourne, the company supplying the EPD vials, and to no one else. Prior to delivery of vials to London, they labelled the identical vials with sequential numbers (1–50) according to the allocation sequence and placed them in two boxes (one for the first and one for the second injection). There was no further contact between the laboratory and any of the investigators for the duration of the trial. After enrolment and first mosquito exposure by N. Hill, S. Berkovitz allocated the next available number at each subject's first EPD injection. The code was only revealed to the researchers once recruitment and data collection were complete, and data analysis was proceeding. All study personnel and participants were not aware of treatment assignment throughout the study. However, participants may have become inadvertently unblinded because of prolonged swelling or itching at the site of injection after EPD compared to placebo at 24 hours. This was explored using a participant questionnaire filled out at 24 hours after each injection and returned by post to the investigators.

## 3. Results

**Figure 1:**
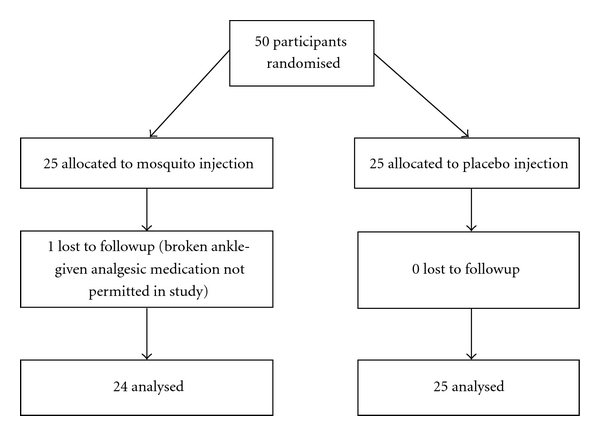
Participant flow.

Participant flow is shown in Figure 1. One patient in the mosquito injection group was lost to followup; thus data from 49 participants were available for the intention-to-treat analysis. Therefore, all participants who underwent random allocation were analyzed according to group assignment. One other patient violated the protocol as they had received the wrong vial in error but, since both vials were placebo, this was thought unlikely to affect the results. Baseline data are displayed in Table 1, showing good comparability between groups.

Primary outcomes are summarised in Table 2. At 1 hour following bite, mean SREA was slightly higher in the EPD group compared to the placebo group after adjusting for baseline values (0.46, 95% confidence interval −6.11 to 7.03), but this was not close to statistical significance suggesting little evidence of any effect attributable to EPD (*P* = 0.89, ANCOVA analysis). Both groups decreased slightly compared to their baseline values. Likewise, at 24 hours after bite, mean SREA was slightly lower in the EPD group compared to the placebo group after adjusting for baseline values (−2.58, 95% CI −11.73 to 6.57), but again this was not statistically significant (*P* = 0.57). This time both groups increased compared to their baseline values. The proportion of participants experiencing a decrease in wheal size was similar for EPD and placebo and not one likely to prove clinically useful.

We then restricted the analysis to patients who had exhibited exposure diameters exceeding 50 mm in the baseline measures. There were 23 patients in this subgroup (8 on placebo and 15 on drug). Repeating the ANCOVA analyses for the 1 hour and 24 hour data led to no difference in the conclusions. The estimates were 1 hour: −0.54 (−12.20 to 11.12, *P* = 0.92) and 24 hour: 7.02 (−9.70 to 23.73, *P* = 0.39). Although there is an imbalance (by chance) in the numbers in the subgroup analysis, the ANCOVA does take account of baseline measures.

For the secondary outcome, that of itching at the time of controlled bite and 24 hours afterwards, the results are given in Table 3. A larger proportion of participants experienced a reduction in itching at the time of bite with EPD, whereas at 24 hours after bite there was a larger proportion with placebo. In neither case was there a statistically significant difference between groups.

Success of blinding was indirectly estimated by analysing the proportion of participants who noticed itch or swelling associated with the injection. For the EPD participants, 41/50 injections produced swelling noticeable to the participants 24 hours after the injection, with a mean diameter (patient-measured using a ruler) of 6.4 mm. For the placebo participants, only 11/48 injections produced such a reaction, with a mean diameter of 8.5 mm. This might indicate some degree of unblinding, since persistent swelling might lead participants to conclude (correctly) that they had received verum. One participants' data was missing. However, only two participants complained of itching at 24 hours, both in the placebo group.

Only two adverse effects occurred other than expected local reactions. Both of these occurred in the placebo group. One patient sustained myalgia lasting a few hours; the other had a mild vasovagal episode 10 minutes following the injection.

## 4. Discussion

The disappointing results contrast with a retrospective audit of 11 practitioners carried out by the first author prior to the study. This documented 53 patients, of whom 83% were said to have achieved a moderate or great improvement after two EPD injections of mosquito antigen. The most likely reason for the discrepancy is observer bias in everyday practice. Another possible but unlikely reason is that we gave injections at 6 weekly intervals (due to time pressure), whereas the recommended interval is 8–12 weeks. Since the native mosquito extract was not analysed or standardised for allergen content, the exact amount of administered antigen is unknown and likely to show batch-to-batch variation. Therefore, it is possible that a higher dose might have produced a better clinical effect, although by definition EPD is a “low-dose” treatment.

**Figure 2:**
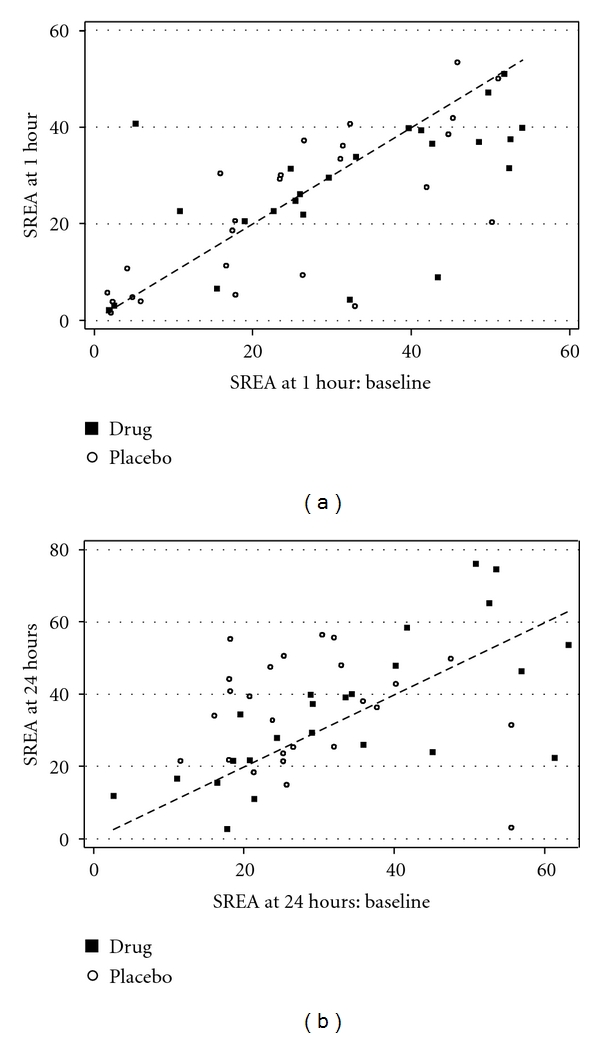
Scatter plots illustrating individual participants' results. (a) Results at one hour after mosquito bite exposure. (b) Results at 24 hours after mosquito bite exposure.

A noteworthy feature of the controlled mosquito bites is the high variation between participants suggesting that a cross-over trial might be appropriate for future studies in this area. Also, there is relatively high intraparticipant variability in the placebo group over a few months, with erythema diameters varying by a factor of up to 2 and therefore erythema area by a factor of up to 4. Scatter plots (Figures 2(a) and 2(b)) illustrate both the sources of variation and suggest that relatively large numbers of patients would be required for a definitive study, had the results of our pilot showed a trend towards efficacy (which was not the case).

## 5. Conclusion

This pilot study suggests that EPD, although safe, is not of value in preventing large local reactions to mosquito bites or the itching associated with them, and that a larger study would not be worth performing. Methodological problems to note include a high variability in LLR-MB across subjects, suggesting that a cross-over trial design should be used for further studies of this condition.

## Figures and Tables

**Table 1:** Baseline data.

Characteristic	Mosquito injection group (*n* = 25)	Placebo group (*n* = 25)
Mean age (±SD)	37.26 (8.32)	37.17 (8.23)
Sex (F/M)	19/5	22/3
History of atopic disease	12	12
Previous desensitization	2	1
On regular medication	6	10

Perceived severity of previous reactions (0–3; 0 = mild, 3 = severe)	0 0	0 0
	1 1	1 1
	2 19	2 19
	3 3	3 2
	Missing data 2	Missing data 3

Perceived duration of previous reactions (1–5; 1 = less than an hour, 5 = greater than 24 hours)	1 0	1 1
	2 0	2 3
	3 1	3 1
	4 4	4 5
	5 19	5 16
	Missing data 1	

**Table 2:** Results for primary outcome measure, the mean square root of erythema area (SREA) at 1 hours and 24 hours post-bite.

Outcome	Mean (SE) of SREA (mm) Mosquito antigen (*n* = 24)	Mean (SE) of SREA (mm) Placebo (*n* = 25)	Difference in mean SREA (drug-placebo)* (95% CI)	*P*
Baseline				
1 hour	31.3 (3.4)	24.5 (3.2)		
24 hours	33.7 (3.4)	28.7 (2.3)		
Followup				
1 hour	27.4 (2.9)	22.6 (3.2)	0.46 (−6.11 to 7.03)	0.89
24 hours	35.1 (2.4)	35.0 (2.9)	−2.58 (−11.73 to 6.57)	0.57
Proportion of participants with a decrease in SREA at				
1 hour	14/24 (58%)	12/25 (48%)		
24 hours	8/24 (33%)	9/25 (36%)		

*Adjusted for baseline values (ANCOVA analysis).

**Table 3:** Results for secondary outcome measure, change in itching at time of bite and 24 hours afterwards.

Proportion of participants with a reduction in itching	EPD	Placebo	P*
Time of bite	9/24 (38%)	13/25 (52%)	0.31
24 hours after bite	14/24 (58%)	11/25 (44%)	0.32

*Chi-squared test.

## Abbreviations

EPD: Enzyme potentiated desensitisation
LLR-MB: Large local reactions to mosquito bites
SREA: Square root of erythema area.
